# A non-linear partial least squares based on monotonic inner relation

**DOI:** 10.3389/fphys.2024.1369165

**Published:** 2024-05-01

**Authors:** Xuepeng Zheng, Bin Nie, Jianqiang Du, Yi Rao, Huan Li, Jiandong Chen, Yuwen Du, Yuchao Zhang, Haike Jin

**Affiliations:** ^1^ School of Computer, Jiangxi University of Chinese Medicine, Nanchang, China; ^2^ National Pharmaceutical Engineering Center for Preparation of Chinese Herbal Medicine, Jiangxi University of Chinese Medicine, Nanchang, China

**Keywords:** Chinese medicine, drug dose-effect relationship, partial least squares, non-linear modeling, monotonic cubic splines

## Abstract

A novel regression model, monotonic inner relation-based non-linear partial least squares (MIR-PLS), is proposed to address complex issues like limited observations, multicollinearity, and nonlinearity in Chinese Medicine (CM) dose-effect relationship experimental data. MIR-PLS uses a piecewise mapping function based on monotonic cubic splines to model the non-linear inner relations between input and output score vectors. Additionally, a new weight updating strategy (WUS) is developed by leveraging the properties of monotonic functions. The proposed MIR-PLS method was compared with five well-known PLS variants: standard PLS, quadratic PLS (QPLS), error-based QPLS (EB-QPLS), neural network PLS (NNPLS), and spline PLS (SPL-PLS), using CM dose-effect relationship datasets and near-infrared (NIR) spectroscopy datasets. Experimental results demonstrate that MIR-PLS exhibits general applicability, achieving excellent predictive performances in the presence or absence of significant non-linear relationships. Furthermore, the model is not limited to CM dose-effect relationship research and can be applied to other regression tasks.

## 1 Introduction

Predicting the dose-effect relationship of Chinese Medicine (CM) is crucial for the modernization of CM. To avoid costly and laborious, yet not always deterministic, experiments for determining dose-effect relationships, novel and efficient prediction approaches are needed. However, CM data differ significantly from other types of data. In dose-effect relationship experiments, due to multiple active ingredients in drugs and limited experimental trials, the data often exhibit characteristics where the number of samples is less than the number of variables or not significantly larger than the number of variables. Additionally, the dose-effect relationship can be both linear and non-linear. Therefore, multicollinearity and nonlinearity may exist in the experimental data.

Partial least squares (PLS) (Wold et al., 1982) has been proven to be an effective regression method for dealing with noise-corrupted and highly correlated data with limited observations (Frank and Friedman, 1993) and is widely applied in various fields, including chemometrics (Wold et al., 2001), econometrics (Korkmazoglu and Kemalbay, 2012), bioinformatics (Nguyen and Rocke, 2002), medicine (Worsley, 1997), and pharmacology (Bro, 1996), *etc.* However, while PLS can address issues like multicollinearity, it may result in poor predictive performance in the presence of a non-linear relationship between **X** and **Y**. To address this limitation, researchers have developed various non-linear PLS (NL-PLS) modeling approaches.

One major approach for NL-PLS modeling is based on non-linear iterative partial least squares (NIPALS) algorithm (Wold, 1975). The fundamental idea is to retain the outer model and replace the linear inner model with a non-linear function within the standard PLS framework. Qin and McAvoy, (1992) proposed a neural network PLS (NNPLS) algorithm, which uses artificial neural networks (ANN) to capture the inner relations. However, since the weight updating strategy (WUS) of NIPALS does not consider non-linear relationships, such modifications may lead to poor predictive performance (Baffi et al., 2000; Searson et al., 2007). Wold et al. (1989) developed a quadratic PLS (QPLS) algorithm, which further modifies the NIPALS algorithm, addressing how to update the projection coefficients (the weights w) of the input matrix while considering the non-linear relationship. Baffi et al. (1999) presented an error-based quadratic PLS (EB-QPLS) algorithm based on Wold’s original work, which modifies and simplifies the WUS of QPLS. However, EB-QPLS often encounters over-fitting when handling multicollinear data. Subsequently, Shan et al. (2015) introduced a nested PLS structure for further improvement. This strategy adopts standard PLS to replace multiple linear regression (MLR) in the WUS of EB-QPLS, effectively addressing the multicollinearity problem. Furthermore, Wold, (1992) provided another WUS based on the covariance criterion and proposed the spline PLS (SPL-PLS) algorithm.

Several state-of-the-art methods for analyzing the dose-effect relationship of CM through NL-PLS have been developed. Nie et al. (2023) proposed a novel regression method called partial least distance squares (PLDS), which reflects the original data information through distance variance, measures the correlation between input and output scores using the distance correlation coefficient, and constructs the final regression equation using a quasi-linear regression method. Xiong et al. (2020) utilized a deep belief network (DBN) to extract upper-level features from the original data and applied them to the linear PLS model. Xiong et al. (2023) proposed an analytical model that combines deep Boltzmann machine (DBM) and PLS to address the challenge of dose-effect relationship analysis. This method, which maps raw data to a new representation (data space) using a non-linear function and applies linear PLS, is another major approach in NL-PLS modeling.

For the above NL-PLS models, there are some inherent drawbacks. For example, the method of directly using ANN to extract non-linear features, although it can transform the features of the original data, also leads to the over-parameterized problem (Qin and McAvoy, 1992). The performance of NL-PLS based on the NIPALS algorithm mainly depends on the fitting effectiveness of the inner model and the WUS. If the approximation capability of the inner model is very limited, it cannot provide enough flexibility to model complex non-linear inner relations. Conversely, excessive flexibility may lead to issues such as over-fitting and local minima (Shan et al., 2015). Moreover, the WUS also has a large impact on predictive performance.

In the study of the dose-effect relationship of CM, the existing NL-PLS algorithms may excel in specific scenarios but lack general applicability. Inspired by the aforementioned NL-PLS algorithms, we proposed a novel NL-PLS algorithm named monotonic inner relation-based PLS (MIR-PLS). In this methodology, non-linear relationships are iteratively modeled using monotonic cubic spline piecewise regression, which reduces the risk of over-fitting. Additionally, by leveraging the property that the inverse of a monotonic function is unique, we developed a new WUS to improve predictive performance. MIR-PLS was compared with standard PLS, QPLS, EB-QPLS, NNPLS, and SPL-PLS on CM dose-effect relationship datasets and two near-infrared (NIR) spectroscopy datasets. The Wilcoxon signed rank test was employed to determine whether the predictive performance of MIR-PLS significantly differed from that of the other models. The results demonstrated that our MIR-PLS algorithm has better predictive performances than other PLS algorithms, making it a reliable and robust regression tool in the presence or absence of significant **X-Y** non-linear relationships.

## 2 Related works

### 2.1 Linear PLS algorithm

Linear PLS regression is a special algorithm that integrates MLR analysis, canonical correlation analysis, and principal component analysis. It can effectively deal with correlated input and limited data. Given an input data matrix $X\in R^{N\times M}$ and an output data matrix $Y\in R^{N\times K}$ , the PLS model first decomposes the matrices **X** and **Y** into bilinear products plus residual matrices:

$$X={\text{TP}}^{T}+E=\sum_{h=1}^{A}t_{h}p_{h}^{T}+E$$ (1)

$$Y={\text{UQ}}^{T}+F=\sum_{h=1}^{A}u_{h}q_{h}^{T}+F$$ (2)

where *A* represents the number of latent variables (LVs); $T=\left[t_{1},...,t_{A}\right]\in R^{N\times A}$ and $U=\left[u_{1},...,u_{A}\right]\in R^{N\times A}$ are the score matrices of **X** and **Y**, respectively; $t_{h}\in R^{N\times 1}$ and $u_{h}\in R^{N\times 1}$ are the score vectors of **X** and **Y**, respectively; $P=\left[p_{1},...,p_{A}\right]\in R^{M\times A}$ and $Q=\left[q_{1},...,q_{A}\right]\in R^{K\times A}$ are the loading matrices of **X** and **Y**, respectively; $p_{h}\in R^{M\times 1}$ and $q_{h}\in R^{K\times 1}$ are the loading vectors of **X** and **Y**, respectively; and **E** and **F** are the corresponding residual matrices.

Eqs 1, 2 formulate a PLS outer model. After the outer model is constructed, the input and output score vectors are related by a linear inner model:

$$u_{h}=b_{h}t_{h}+e_{h},h=1,2,...,A$$ (3)

where $b_{h}$ is a regression coefficient which is determined by minimizing the residuals $e_{h}$ .

*A* is usually determined through cross-validation. Theoretically, if more LVs are kept, the less information is left in the residual matrices **E** and **F**, and the better the model fits the training samples. However, training the model in this way often leads to over-fitting. Cross-validation avoids over-fitting and ensures the model’s generalization to unseen data. We recommend referring to the literature (Geladi and Kowalski, 1986; Höskuldsson, 1988; Martens and Naes, 1992) for readers interested in the detailed PLS algorithm.

### 2.2 NL-PLS algorithms

The linear PLS is constrained by its linear inner model. Given the prevalence of non-linear data, there is a compelling need to develop a non-linear counterpart that retains the robust attributes of linear PLS. Adhering to the linear PLS framework, the development of NL-PLS models is straightforward. This involves retaining the linear outer model while substituting the linear inner relation with a non-linear function, resulting in a modified form of Eq. 3:

$$u=\phi_{NL}\left(t\right)+e$$ (4)

where $\phi_{NL}\left(\cdot \right)$ is the nonlinear function. In this way, there are countless possible non-linear extensions of linear PLS. However, the introduction of a non-linear relationship also makes the input weights **w** difficult to calculate.

## 3 Methodology

Any iterative NL-PLS algorithm involves two major modifications to the original NIPALS algorithm: weights (**w**) updating and a non-linear model between the input and output scores. Our proposed algorithm has two distinctive characteristics. Firstly, it utilizes a piecewise regression function based on a monotonic cubic spline to model the relationships between **t** and **u**. Secondly, its WUS is based on the properties of monotonic functions and the idea of nested PLS. The following sections focus on predicting a single variable **y** from a multivariate matrix **X** and provide a detailed explanation of the MIR-PLS algorithm.

### 3.1 Monotonic inner model

Wold, (1992) first proposed the NL-PLS method using a regression spline to build the inner model. Splines offer flexibility to model complex non-linear relationships, providing smooth and continuous fits to the data. They reduce over-fitting by dividing the data into smaller pieces and allow control over model complexity through the number and placement of knots (Wold, 1974; Wegman and Wright, 1983; Friedman, 1991).

Although Wold proposed using linear, quadratic, and cubic splines to construct the inner model, in practice, cubic splines are sufficient to approximate any continuous function. Cubic splines can be composed of multiple pieces, where a cubic polynomial is fitted to the data points in each piece. To ensure a smooth and continuous curve, the polynomial pieces are connected at knots, and continuity constraints are imposed on the function and all its derivatives except the highest order (third order for cubic splines), which endows the splines with high flexibility and approximation power. The bivariate cubic polynomial with **u** as the dependent variable and **t** as the predictor variable, denoted as $u=S\left(t\right)$ , is expressed as:

$$u=b_{0}+b_{1}t+b_{2}t^{2}+b_{3}t^{3}$$ (5)

It has been demonstrated that modeling non-linear relationships using piecewise cubic splines is a flexible and reliable regression method (Durrleman and Simon, 1989). However, if the number of segments used in the cubic splines is too high, the risk of over-fitting can increase. In addition, piecewise fitting may not fully consider the overall distribution characteristics of the data, leading to poor predictive performances in the presence of noise or random fluctuations. Therefore, we propose imposing monotonicity constraints on the cubic splines, as monotonicity constraints have been proven to reduce the risk of over-fitting (Fritsch and Carlson, 1980; Sill and Abu-Mostafa, 1996). A cubic spline imposing monotonicity constraints can be expressed as:

$$u=b_{0}+b_{1}t+b_{2}t^{2}+b_{3}t^{3},\;s.t.du/dt\geq 0$$ (6)

**FIGURE 1:**
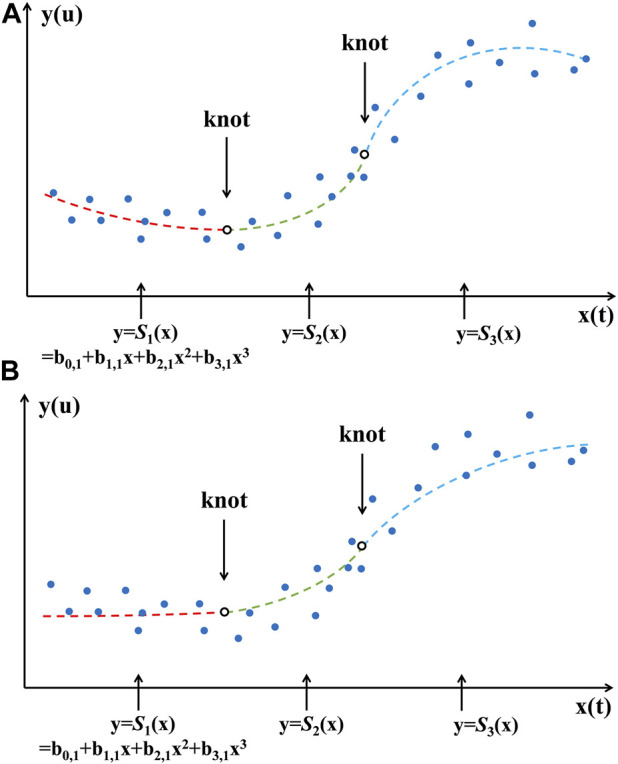
Cubic spline **(A)** and monotonic cubic spline **(B)** with two knots.

Monotonic cubic spline piecewise regression retains flexibility while adequately considering the overall data distribution. Figure 1 shows cubic and monotonic cubic splines with two knots, respectively.

The free parameters in splines are the places and number of knots. In the current modeling, the placement of knots will not be considered as free parameters. In this study, we will make the number of data points in each polynomial piece as equal as possible based on the number of knots. The procedure involves sorting the dataset based on the independent variable’s values, determining the desired number of segments (and thus the number of knots), and then dividing the total number of data points by the number of segments to estimate the data points per segment. Knots are positioned at intervals in the sorted data corresponding to these segment boundaries. For example, with 100 data points and a goal of four segments, we would place knots at the end of every 25th data point, resulting in segments of roughly equal size. This approach ensures a uniform distribution of data points across the spline segments, enhancing the model’s robustness and interpretability.

The flexibility of splines is influenced by the number of knots, and increasing their number also increases the risk of over-fitting. Some researchers have suggested that five knots should be sufficient for modeling the most common monotonic non-linear relationships while minimizing the risk of over-fitting (De Boor and De Boor, 1978). In practical applications, it is advisable to estimate the number of knots using cross-validation.

While the monotonicity constraint helps reduce the over-fitting risk, it may also decrease fitting accuracy. Thus, we introduce a novel approach to update the **X** weights (**w**), which will be explained in the next section.

### 3.2 Weight updating strategy

In many iterative NL-PLS algorithms, the weights calculation considers the non-linear relationship. In our proposed method, the non-linear relationship is also taken into account, but in a more direct way.

In linear PLS, the **t** scores are proportional to the **u** scores, which means that for any u, there is a unique corresponding t. Non-linear models with monotonicity constraints preserve this property, allowing fully reversible non-linear relationships:

$$u=\phi_{NL}\left(t\right),\;t=\phi_{NL}^{-1}\left(u\right)$$ (7)

This property enables more convenient incorporation of the non-linear relationship $\phi_{NL}\left(\cdot \right)$ when calculating weights **w**. In the NIPALS algorithm, the loading vector **q** for **y** is calculated using the predicted score vector $\hat{u}$ , i.e., $q^{T}={\hat{u}}^{T}y/{\hat{u}}^{T}\hat{u}$ . Then **q** is used to update **u** scores, i.e., u=yq . At this point, the updated **u** scores already encompass the non-linear relationship $\phi_{NL}\left(\cdot \right)$ . In MIR-PLS, the predicted input score vector $\hat{t}$ can be calculated using the updated **u** and Eq. 7, i.e., $\hat{t}=\phi_{NL}^{-1}\left(u\right)$ . Subsequently, the error **e** between $\hat{t}$ and **t** can be expressed as:

$$e=\hat{t}-t=X\hat{w}-\text{Xw}=X\Delta w$$ (8)

According to the above equation, the weight corrections Δw can be computed by regressing **e** on the input matrix **X**. However, in the case of dose-effect and spectroscopic datasets, **X** tends to exhibit high collinearity. The multicollinearity problem will hinder WUS from providing precise input weights, leading to the risk of over-fitting. To solve this problem, we draw inspiration from nested PLS (Li et al., 2005). Specifically, we establish a regression model using standard PLS and employ the obtained Δw as the regression coefficients:

$$\Delta w=\text{PLS}\left(X,e\right)$$ (9)

For nested PLS, the number of LVs included within the inner PLS algorithm can be determined through cross-validation. However, built-in cross-validation may increase the training cost of the MIR-PLS model. To strike a balance between prediction accuracy and computational efficiency, we selected two LVs for the internal PLS, accepting a trade-off that may not yield optimal prediction but does enhance practicality.

The weight corrections will be utilized to update the input weights **w**:

$$w^{new}=w+\Delta w$$ (10)

Eqs 8 to 10 lead to a new WUS, which directly involves the non-linear relationship $\phi_{NL}\left(\cdot \right)$ in calculating the corrections Δw and adopts the nested PLS strategy. This methodology ensures robust and accurate input weights while reducing over-fitting risks. Algorithm 1 provides a detailed description of the steps of the MIR-PLS algorithm and its input weights updating process.

Algorithm 1 **The MIR-PLS Algorithm.**

1. Scale **X** and **y** to zero-mean and one-variance;
2. Set *A* factors;
3. Let h=1 , $X_{h}=X$ and $y_{h}=y$ ;
4. **while** h≤A **do**:
5. Let $u_{h}=y_{h}$ ;
6. Calculate the input weight vector $w_{h}^{T}={u_{h}^{T}X_{h}/u_{h}^{T}u}_{h}$ and normalize it to length 1.0;
7. Calculate the input score vector $t_{h}=X_{h}w_{h}$ ;
8. Set i=0 ;
9. **while** i<30 **do**:
10. Estimate ${\hat{u}}_{h}=\phi_{NL}\left(t_{h}\right)$ by the monotonic inner relation;
11. Calculate the output loading vector $q_{h}^{T}={\hat{u}}_{h}^{T}y_{h}/{\hat{u}}_{h}^{T}{\hat{u}}_{h}$ and normalize it to length 1.0;
12. Update output scores $u_{h}=y_{h}q_{h}$ ;
13. Estimate ${\hat{t}}_{h}=\phi_{NL}^{-1}\left(u_{h}\right)$ by the reversible relation;
14. Calculate the weight corrections $\Delta w_{h}=\text{PLS}\left(X_{h},{\hat{t}}_{h}-t_{h}\right)$ ;
15. Update the input weight vector $w_{h}^{new}=w_{h}+\Delta w_{h}$ and normalize it to length 1.0;
16. Update the input scores $t_{h}^{new}=X_{h}w_{h}^{new}$ ;
17. **if** $\left\|t_{h}^{new}-t_{h}\right\|/\left\|t_{h}\right\|<10^{-6}$ **then**
18. **break**
19. **end if**
20. i++

21. **end while**
22. Calculate the input loading vector $p_{h}^{T}=t_{h}^{T}X_{h}/t_{h}^{T}t_{h}$ ;
23. Update ${\hat{u}}_{h}^{new}=\phi_{NL}\left(t_{h}^{new}\right)$ ;
24. Calculate the residual matrices $X_{h+1}=X_{h}-t_{h}p_{h}^{T}$ and $y_{h+1}=y_{h}-{\hat{u}}_{h}^{new}q_{h}^{T}$ ;
25. h++ ;
26. **end while**

## 4 Experiments

In the experimental studies, the performances of MIR-PLS were first validated using the CM dose-effect relationship datasets, characterized by the presence of linear, non-linear, and correlated inputs, and the number of samples is not significantly larger than the number of variables. Additionally, two NIR datasets were used to demonstrate the generality of our proposed algorithm. The Wheat kernel dataset is a typical non-linear dataset. In the Beer dataset, **X** contains a large number of variables compared to the number of observations.

### 4.1 Dataset description

#### 4.1.1 Dose-effect relationship datasets of Chinese Medicine

CM datasets with dose-effect relationships were used to evaluate the performances of different models. These datasets include three sets of data on Maxingshigan Decoction (MXSGD) in the treatment of cough, asthma, and fever. They are called anti-tussive (AT) model data, anti-asthmatic (AA) model data, and anti-febrile (AF) model data, respectively. They were obtained from two subjects, including Study on Dose-effect Relationship of Traditional Chinese Medicine (TCM) Prescription based on Pharmacodynamic Substances (Subject No.2010CB530602) and Study on Dose-effect Relationship of TCM Prescription based on Pharmacodynamics (Subject No. 2010CB530603).

In the experimental study of the dose-effect relationship of MXSGD in the treatment of cough, 12 different ratios of the formula were prepared. Each ratio was used to treat ten diseased rats, and then plasma concentration and pharmacological indicators were measured for each rat and averaged to obtain a sample. The concentrations of drug components (including Ephedrine, Pseudo-ephedrine, Methylephedrine, Amygdalin hydrate, Prunasin, Liquiritin, Liquiritigenin, and Glycyrrhetinic acid) in blood represent predictor variables, and the pharmacological indicator (the frequency of coughs) represents response variable. Therefore, in the AT dataset, the number of samples *N* = 12, the dimension of the input vector *M* = 8, and the dimension of the output vector *K* = 1.

In the AA dataset, 13 different ratios were prepared. The predictor variables are the same as the AT dataset, and the response variables are the incubation period (s) and the duration (min) of asthma. Therefore, the number of samples *N* = 13, the dimension of the input vector *M* = 8, and the dimension of the output vector *K* = 2.

In the AF dataset, 13 different ratios were prepared. The predictor variables are the same as the AT dataset, and the response variables are PGE2, TRI temperature index, and 6-h fever inhibition rate. Therefore, the number of samples *N* = 13, the dimension of the input vector *M* = 8, and the dimension of the output vector *K* = 3.

The data analysis task in this project is to establish a model between plasma concentration and pharmacological indicators to predict the effect of drug concentration in the blood on pharmacological actions. These three datasets are typical examples where the number of samples is not significantly larger than the number of variables. Further descriptions and modeling based on these datasets can be found in References (Xiong et al., 2020; Nie et al., 2023; Xiong et al., 2023). The specific descriptions of the three datasets are shown in Table 1.

**TABLE 1:** The specific descriptions of dose-effect relationship datasets.

Datasets	Number of predictor variables	Number of response variables	Number of samples
AT	8	1	12
AA	8	2	13
AF	8	3	13

#### 4.1.2 Wheat kernel dataset

The Wheat Kernel dataset is related to the NIR transmittance spectra of the wheat kernel. The nonlinearity of this dataset partly came from the light scatter effect. The calibration set has *N* = 415 samples of *M* = 100 wavelengths (850–1,050 nm at intervals of 2 nm). One response variable yields the protein concentration. The test set is composed of 108 spectra of wheat kernels. Further descriptions and modeling based on this dataset can be found in References (Helland, 2001; Shan et al., 2015).

#### 4.1.3 Beer Dataset

This dataset (Beer Data) contains *N* = 80 samples of *M* = 576 wavelengths (1,100–2,250 nm with a 2 nm interval) published by Norgaard et al. (2000). One response variable is the measured “original extract concentration.” This is an important quality parameter in the brewing industry, indicating the substrate potential for the yeast to ferment, giving rise to higher alcoholic content. The dataset is split into two sets, taking every third sample (i.e., 0, 3, … , 78) as a test set and the remaining samples as the calibration set.

### 4.2 Evaluation metrics

To evaluate the predictive performances of different calibration models, the root mean square error (RMSE), the mean absolute percentage error (MAPE), and the coefficient of determination (*R* ^2^) were utilized and defined as

$$\text{RMSE}=\sqrt{{\left(\hat{y}-y\right)}^{T}\left(\hat{y}-y\right)/N}$$ (11)

$$\text{MAPE}=\frac{1}{N}\times \sum_{i=1}^{N}\left|\left(y_{i}-{\hat{y}}_{i}\right)/y_{i}\right|\times 100\%$$ (12)

$$R^{2}=1-\frac{\sum_{i=1}^{N}{\left(y_{i}-{\hat{y}}_{i}\right)}^{2}}{\sum_{i=1}^{N}{\left(y_{i}-\bar{y}\right)}^{2}}$$ (13)

where y is the vector of the response variable, $\hat{y}$ is the vector of the predicted response variable, and *N* is the number of samples. $y_{i}$ and ${\hat{y}}_{i}$ are the i th elements of y and $\hat{y}$ , respectively. $\bar{y}$ denotes the mean of the response variable.

For each dose-effect relationship dataset, it was not divided into a train set and test set due to the limited number of samples. Therefore, we used leave-one-out cross-validation (LOO-CV) (Stone, 1974; Oner et al., 2021) for parameter tuning and calculated the RMSE, the MAPE, and the R^2^ to estimate the prediction accuracy for each model. To demonstrate the RMSE improvement of MIR-PLS better, a parameter from Reference (Shan et al., 2015) was introduced as

$$k=\left(1-\frac{{\text{RMSE}}_{\text{MIR}-\text{PLS}}}{{\text{RMSE}}_{\text{other}}}\right)\times 100\%.$$ (14)

For the two NIR datasets, the calibration set was utilized for model building, involving both training models and performing 5-fold cross-validation for parameter tuning. RMSE of calibration (RMSEC), RMSE of cross-validation (RMSECV), and MAPE of cross-validation (MAPECV) were calculated in these steps. Subsequently, the test set was employed to calculate the RMSE of prediction (RMSEP) and R^2^ to assess the predictive performances of the trained model. The *k* value of RMSEP improvement was calculated as

$$k=\left(1-\frac{{\text{RMSEP}}_{\text{MIR}-\text{PLS}}}{{\text{RMSEP}}_{\text{other}}}\right)\times 100\%.$$ (15)

The Wilcoxon signed rank test (Wilcoxon, 1992) was employed at a 95% confidence level to determine whether a statistically significant difference exists between the two competing models. This evaluation was conducted by comparing the prediction errors of the two different models.

### 4.3 Parameter tuning

The predictive performances of PLS, QPLS, EB-QPLS, NNPLS, SPL-PLS, and MIR-PLS rely on the tuning of model parameters. Using cross-validation to select model parameters can help alleviate over-fitting and improve the generalization ability of the trained model (Wold, 1978; Osten, 1988). In this paper, we selected parameters based on the minimum RMSECV value. The optimal number of LVs, an essential parameter in each model, was estimated within the range of [1,15].

For the NNPLS method, an additional vital parameter is the optimal number of hidden units for each LV. This parameter was automatically determined by the formula defined as

$$\tau =\frac{1}{N}\times \sum_{i=1}^{N}\left|{\hat{u}}_{i}\left(nn+1\right)-{\hat{u}}_{i}\left(nn\right)\right|$$ (16)

where nn represents the number of hidden layer neurons at the current LV and ${\hat{u}}_{i}\left(nn\right)$ is the i th element of the predicted output score vector. When the τ value is less than the predefined threshold (0.01), it indicates that adding a new hidden unit does not significantly reduce the fitting error and should be discarded. Conversely, the procedure for adding a hidden unit will continue.

For the MIR-PLS method, two crucial parameters were considered: the number of LVs and the number of knots. To optimize these parameters, a grid search with cross-validation was employed. Specifically, the number of LVs varied within the range of [1,15], and the number of nodes was explored in the interval [0,5]. For each combination of these parameters, cross-validation results were computed, and the set of parameters yielding the smallest RMSECV was identified as the optimal configuration.

As for the SPL-PLS method, there are three parameters to determine: the degree of the polynomial (L), the number of knots (J), and the number of LVs. This paper will use a linear, quadratic, or cubic spline to model the inner relation of SPL-PLS. First, L was fixed, and then the optimal number of LVs and J was determined using grid search with cross-validation. Finally, repeated the process with a different L.

## 5 Results and discussion

### 5.1 Performances on Chinese Medicine datasets

The experimental results of the six algorithms on the dose-effect relationship datasets are shown in Table 2. In the table, RMSE, MAPE, and *R* ^2^ are used to compare the performances of each model. LVs represent the optimal number of latent variables for each model. Others display other optimal parameters used in models. It is worth noting that for NNPLS, the specific number of hidden units for each LV is not provided. This is because NNPLS selects different hidden units for each data subset.

**TABLE 2:** Experimental results for the dose-effect relationship datasets.

Dataset	Criteria	PLS	QPLS	EB-QPLS	NNPLS	SPL-PLS	MIR-PLS
AT	RMSE	3.54	3.63	5.38	3.81	3.50	**3.36**
	MAPE	8.09	8.59	13.18	9.73	8.52	**7.45**
	*R* ^2^	0.43	0.41	0.20	0.35	0.45	**0.51**
	LVs	1	1	1	2	1	1
	Others^a^					(2, 1)^b^	1^c^
AA (y1)	RMSE	15.67	22.02	52.56	**12.65**	13.67	14.89
	MAPE	18.20	19.50	39.66	**15.13**	16.61	18.27
	*R* ^2^	−1.02	−2.98	−21.69	**−0.31**	−0.54	−0.82
	LVs	1	3	1	2	1	2
	Others^a^					(1, 5)^b^	1^c^
AA (y2)	RMSE	0.77	**0.70**	1.96	0.73	0.77	**0.70**
	MAPE	7.31	7.39	16.95	7.35	7.94	**6.91**
	*R* ^2^	0.36	0.66	0.09	0.51	0.36	**0.67**
	LVs	1	1	2	1	1	1
	Others^a^					(3, 1)^b^	4^c^
AF (y1)	RMSE	1.00	0.97	1.48	0.99	0.94	**0.93**
	MAPE	29.09	26.47	50.99	32.30	23.32	**20.27**
	*R* ^2^	0.53	0.56	0.13	0.54	0.59	**0.60**
	LVs	2	3	1	4	2	2
	Others^a^					(1, 2)^b^	1^c^
AF (y2)	RMSE	0.88	1.02	3.90	1.03	**0.82**	1.06
	MAPE	9.68	12.35	47.53	12.18	**9.47**	12.43
	*R* ^2^	0.48	0.30	−1.07	0.29	**0.54**	0.25
	LVs	2	1	1	1	3	1
	Others^a^					(1, 1)^b^	3^c^
AF (y3)	RMSE	12.07	12.46	35.43	12.06	**11.30**	11.37
	MAPE	31.63	**27.24**	94.38	29.91	27.79	28.92
	*R* ^2^	0.32	0.28	−3.49	0.32	**0.40**	**0.40**
	LVs	3	2	1	5	2	1
	Others^a^					(1, 4)^b^	1^c^

The bold value means the best performance among different models.

^a^ Other parameters in the model.

^b^ (L, J) used in SPL-PLS, L denotes the degree of polynomial, and J represents the number of knots.

^c^ Number of knots used in MIR-PLS.

In terms of RMSE and MAPE, the proposed MIR-PLS algorithm outperforms the other five comparison algorithms on three datasets (AT, AA (y2), AF (y1)). On the AA (y2) and AF (y1) data sets, the predictive performance of the non-linear PLS models is better than that of the linear PLS. NNPLS achieves the best predictive results on the AA (y1) dataset. It is worth noting that the performance of linear PLS and linear kernel SPL-PLS algorithms on the AF (y2) and AF (y3) datasets is superior to other non-linear PLS algorithms, indicating the weaker non-linear relations on these two datasets. In terms of *R* ^2^, MIR-PLS performs best on the AT, AA (y2), and AF (y1) datasets, with *R* ^2^ values all exceeding 0.5, indicating that the model can explain more than half of the data variance. Linear models outperform non-linear models on the AF (y2) and AF (y3) datasets. It is worth mentioning that the *R* ^2^ values of all models on the AA (y1) dataset are negative, indicating poor model fitting and possibly unsuitability for data interpretation or mismatch between the model and the data.

**FIGURE 2:**
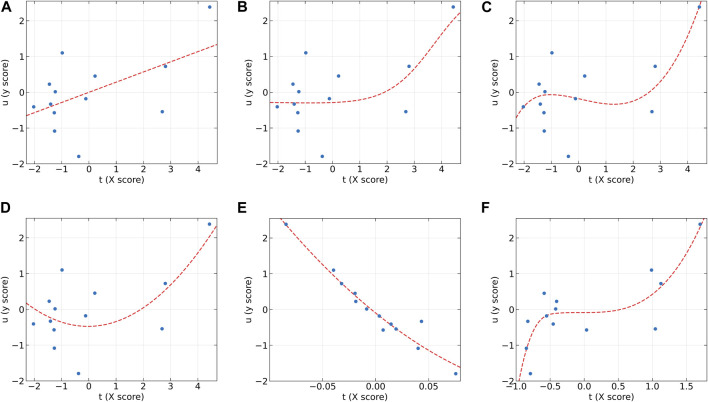
The first LV of the six algorithms on the AA (y2) dataset. **(A)** PLS, **(B)** NNPLS, **(C)** SPL-PLS, **(D)** QPLS, **(E)** EB-QPLS, and **(F)** MIR-PLS.

**FIGURE 3:**
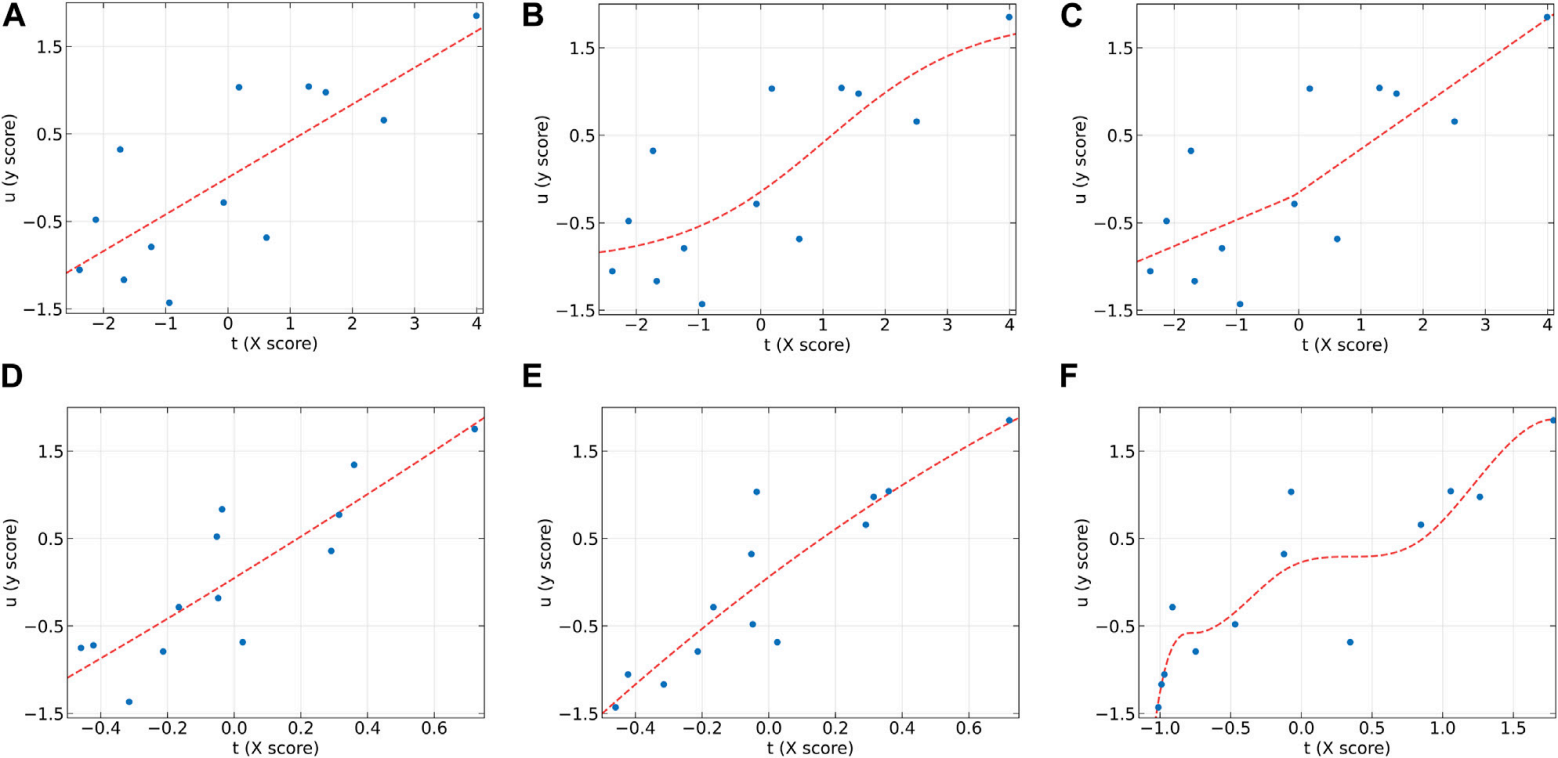
The first LV of the six algorithms on the AF (y2) dataset. **(A)** PLS, **(B)** NNPLS, **(C)** SPL-PLS, **(D)** QPLS, **(E)** EB-QPLS, and **(F)** MIR-PLS.

To further analyze the characteristics of the dose-effect relationship datasets and the differences between these models, the individual inner models of all algorithms at the first LV on the AA (y2) and AF (y2) datasets were plotted in Figure 2, 3, respectively. According to the characteristics of the scatter plots, these six models can be divided into two groups. The first group includes PLS, NNPLS, and SPL-PLS, with almost identical input scores. The second group includes QPLS, EB-QPLS, and MIR-PLS, with a much smaller range of t.

From Figure 2, it can be observed that our proposed MIR-PLS algorithm presents much better predictive performance on the AA (y2) dataset. In Figure 2F, the input scores t obtained by the MIR-PLS projection have a smaller distribution range, and the observed values (blue dots) are close to the non-linear relationship (red dotted line). This indicates that MIR-PLS can better capture the inner relations. Figures 2A–D demonstrate that PLS, NNPLS, SPL-PLS, and QPLS have comparable predictive performances. It is worth noting that EB-QPLS exhibits good flexibility in capturing non-linear inner relations (Figure 2E). However, in terms of predictive metrics, its predictive performance is not ideal, indicating that the extra flexibility may lead to over-fitting. Additionally, the R^2^ values of EB-QPLS are poor across all datasets, with three datasets even showing negative values. This suggests poor model fitting, possibly only predicting the mean of the target variable.

From Figure 3, it can be seen that the linear models seem to perform well. On the AF (y2) dataset, the inner relations (red dotted line) calculated by all algorithms are linear across almost the entire domain. This also confirms that the dataset exhibits linear characteristics. Although MIR-PLS shows slightly lower predictive performance than PLS and SPL-PLS, the statistical test results (Table 3) show no significant difference between them. Furthermore, from Figures 3C, F, it can be observed that the piecewise mapping functions are more flexible in fitting the inner relations compared to single mapping functions.

**TABLE 3:** RMSECV improvements and the Wilcoxon signed rank test results.

		MIR-PLS					
		At	AA (y1)	AA (y2)	AF (y1)	AF (y2)	AF (y3)
PLS	*k* (%)	5.00	4.95	9.09	7.62	−20.25	5.73
	*p*	0.850	0.588	0.273	0.480	0.168	0.094
QPLS	*k* (%)	7.36	32.36	−0.45	4.00	−3.91	8.73
	*p*	0.092	0.542	0.583	0.497	0.787	0.305
EB-QPLS	*k* (%)	37.45	71.66	64.23	37.55	72.82	67.89
	*p*	0.092	0.016	0.048	0.017	0.013	0.068
NNPLS	*k* (%)	11.65	−17.74	3.72	6.45	−2.93	5.65
	*p*	0.064	0.191	0.542	0.787	0.839	0.735
SPL-PLS	*k* (%)	3.82	−8.96	9.03	1.51	−28.50	−0.65
	*p*	0.204	0.542	0.033	0.787	0.008	0.244

**FIGURE 4:**
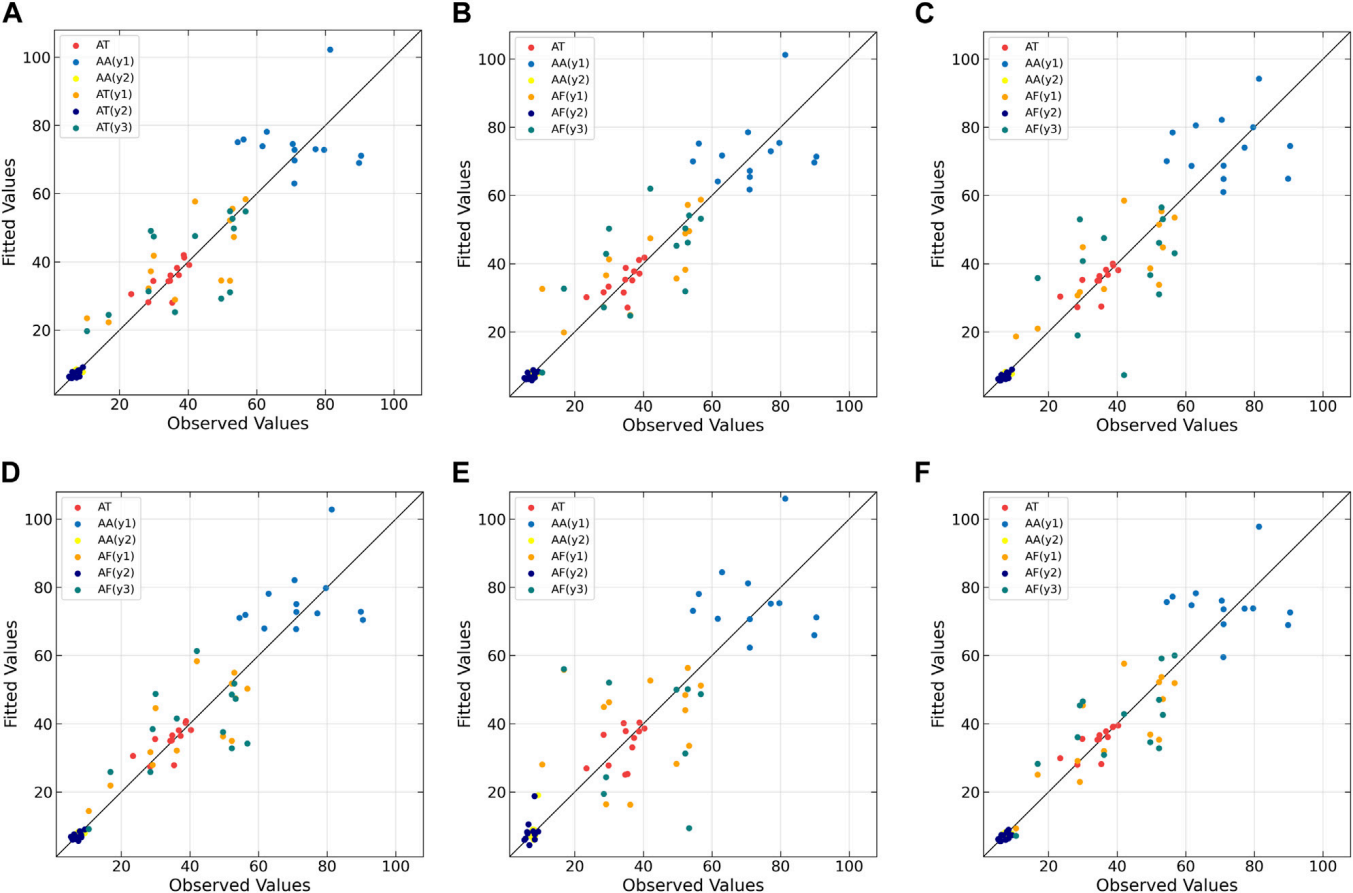
Observed vs. fitted values of response variables for CM datasets as determined by **(A)** PLS, **(B)** NNPLS, **(C)** SPL-PLS, **(D)** QPLS, **(E)** EB-QPLS and **(F)** MIR-PLS.

The correlation between the observed and fitted values is plotted in Figure 4. From the figure, it can be observed that all algorithms do not perform well on the AA (y1) dataset. When dealing with non-linear datasets AT, AA (y2), and AF (y1), the non-linear models exhibit lower predictive errors, whereas linear models show relatively higher. Conversely, on the linear datasets AF (y2) and AF (y3), linear models outperform non-linear models. Although MIR-PLS is not as effective as linear PLS algorithms in handling linear relationships, it still achieves lower predictive errors on the AF (y2) dataset and maintains a good distribution of scatter points near the diagonal line, demonstrating its potential in capturing and predicting linear relationships.

Table 3 presents the improvement of MIR-PLS in terms of RMSE (*k* values). From the table, it can be observed that MIR-PLS enhances the predictive performances on the dose-effect relationship datasets. In addition, the proposed MIR-PLS algorithm requires the fewest LVs on the six datasets. This reflects its ability to capture the latent relationships in the data, and the extracted LVs can effectively interpret the original data. In contrast, other algorithms may require multiple LVs to achieve good predictive performances. However, the Wilcoxon signed rank test indicates that the differences between MIR-PLS and other models are not as significant (except for EB-QPLS). One possible reason is that the sample size is too small to evaluate the differences between the PLS models effectively. Further testing on the NIR datasets will be conducted to examine the differences between PLS models.

In summary, analyzing dose-effect relationship data of Chinese medicine poses significant challenges. No single model can fit all datasets well. In fact, employing single LOO-CV for small datasets may lead to biased estimates of prediction accuracy, as the minimum CV error may not always reflect the lowest test error. In future studies, we aim to employ more rigorous validation techniques, such as double CV, to ensure a more reliable estimate of the predictive performance of our proposed method.

### 5.2 Performances on wheat kernel dataset

Table 4 summarizes the modeling results of the six approaches over the Wheat Kernel dataset. Based on the RMSEP values, the five NL-PLS models outperform the linear PLS model, indicating the inadequacy of linear PLS for this non-linear dataset. In calibration, EB-QPLS achieves the best RMSEC value of 0.446. However, its generalization metrics do not yield the best results. QPLS delivers the second-best RMSEP value, while SPL-PLS presents slightly higher RMSECV and MAPECV compared to MIR-PLS, yet is still competitive. NNPLS, while offering better RMSEP than PLS, does not show better calibration and cross-validation performances. Table 5 shows the number of hidden units used by NNPLS in each LV. Despite not achieving the best calibration performance, MIR-PLS attains the lowest RMSECV and MAPECV values among the six models. MIR-PLS also achieves the best predictive performance, with the lowest RMSEP of 0.556 among the models, further reinforcing its effectiveness in dealing with non-linear datasets. Additionally, MIR-PLS shows the highest *R* ^2^ value at 0.898, suggesting that it not only predicts well but also captures a significant portion of the data’s variability.

**TABLE 4:** Experimental results for the Wheat Kernel dataset.

Criteria	PLS	QPLS	EB-QPLS	NNPLS	SPL-PLS	MIR-PLS
RMSEC	0.505	0.502	**0.446**	0.520	0.492	0.503
RMSEP	0.629	0.577	0.610	0.604	0.620	**0.556**
*R* ^2^	0.869	0.890	0.856	0.880	0.873	**0.898**
RMSECV	0.560	0.558	0.620	0.572	0.549	**0.548**
MAPECV	4.334	4.332	5.008	4.539	4.379	**4.305**
LVs	12	12	2	10	12	12
Others^a^				10^d^	(1, 1)^b^	2^c^

The bold value means the best performance among different models.

^a^ Other parameters in the model.

^b^ (L, J) used in SPL-PLS, L denotes the degree of polynomial, and J represents the number of knots.

^c^ Number of knots used in MIR-PLS.

^d^ The number of hidden units used in NNPLS.

**TABLE 5:** The number of hidden units used in each LV (NNPLS).

Dataset	Number of hidden units									
	1st	2nd	3rd	4th	5th	6th	7th	8th	9th	10th
	LV	LV	LV	LV	LV	LV	LV	LV	LV	LV
Wheat Kernel	1	1	1	1	1	1	1	1	1	1
Beer	5	5	1	1	1	1	1			

**FIGURE 5:**
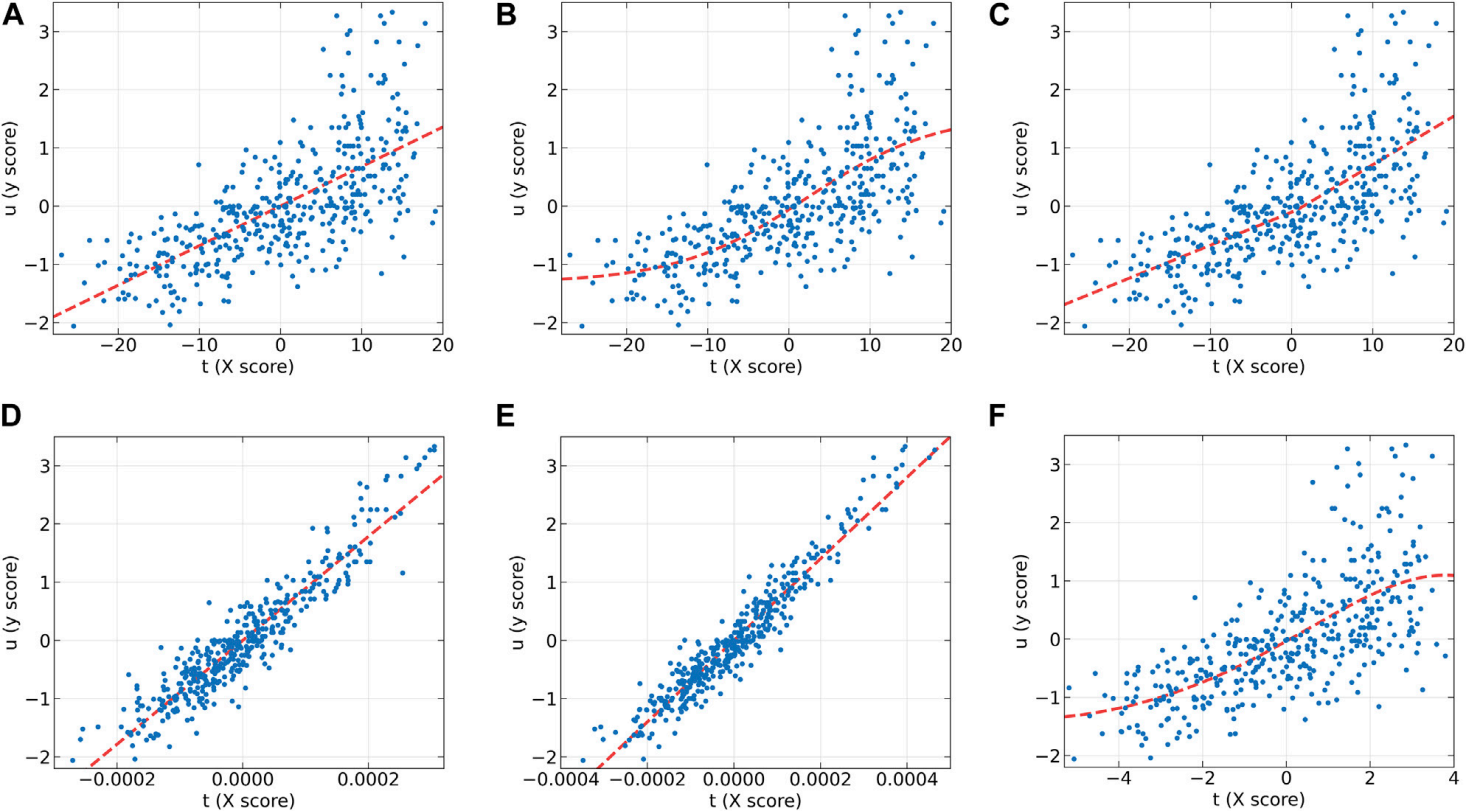
The first LV of the six algorithms on the Wheat Kernel dataset. **(A)** PLS, **(B)** NNPLS, **(C)** SPL-PLS, **(D)** QPLS, **(E)** EB-QPLS, and **(F)** MIR-PLS.

Figure 5 presents the inner relations of all algorithms at the first LV. These plots can easily observe the significant nonlinearity of protein concentration. From Figures 5B, C, it can be seen that NNPLS and SPL-PLS exhibit similar calibration performance. Furthermore, these two models also demonstrate comparable generalization ability. QPLS and EB-QPLS appear to capture the inner relation more effectively (Figures 5D, E). However, EB-QPLS performs slightly better than PLS in terms of RMSEP, indicating potential over-fitting of the inner relation, which hampers further improvement in prediction performance. Conversely, MIR-PLS demonstrates good calibration performance (Figure 5F) while exhibiting excellent generalization performance.

**FIGURE 6:**
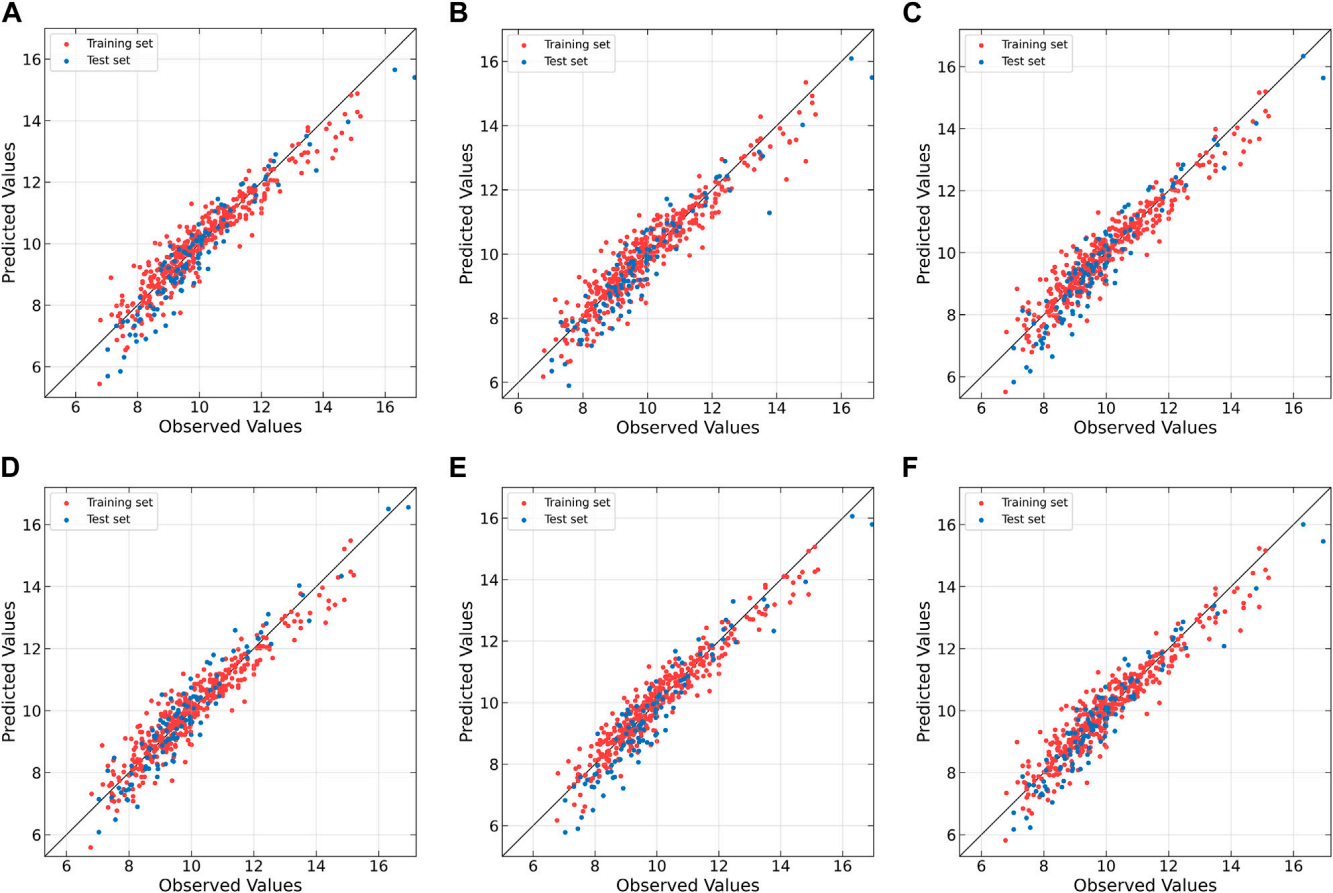
Observed vs. predicted values of protein concentration for Wheat Kernel dataset as determined by **(A)** PLS, **(B)** NNPLS, **(C)** SPL-PLS, **(D)** QPLS, **(E)** EB-QPLS and **(F)** MIR-PLS.

To further compare the predictive performances of different models, the correlation between the observed and predicted values is plotted in Figure 6. From Figure 6A, it can be observed that the correlation is rather poor and the modeling error is relatively high for linear PLS, indicating its inadequacy in handling nonlinearities. The subsequent plots, Figures 6B–E, illustrate improvements in prediction results for NNPLS, SPL-PLS, QPLS, and EB-QPLS, but still deemed unsatisfactory. In contrast, as shown in Figure 6F, MIR-PLS makes the data points align more tightly along the diagonal. The *k* value in Table 6 indicates the enhanced predictive performances of MIR-PLS. Furthermore, the Wilcoxon signed rank test results (Table 6) reveal that the RMSEP value of MIR-PLS significantly differs from other models except for SPL-PLS (*p* = 0.554), further affirming the better prediction performance of MIR-PLS.

**TABLE 6:** RMSEP improvements and the Wilcoxon signed rank test results.

		MIR-PLS	
		Wheat kernel	Beer
PLS	*k* (%)	11.61	11.54
	*p*	0.000	0.594
QPLS	*k* (%)	3.68	28.03
	*p*	0.000	0.141
EB-QPLS	*k* (%)	8.82	70.11
	*p*	0.003	0.485
NNPLS	*k* (%)	7.94	53.51
	*p*	8.1E-15	0.750
SPL-PLS	*k* (%)	10.34	27.43
	*p*	0.554	0.170

### 5.3 Performances on Beer Dataset

In the Beer dataset, the number of predictors (*M* = 576) significantly exceeds the number of samples (*N* = 80). This high dimensionality introduces collinearity problems among the predictors. Building predictive models with algorithms that account for collinearity among predictors considerably reduces the risks of over-fitting. Hence, the Beer dataset presents unique challenges that need to be addressed to ensure accurate modeling and interpretation of the results. Table 7 presents calibration, cross-validation, and prediction performances calculated using different algorithms for the Beer dataset.

**TABLE 7:** Experimental results for the Beer dataset.

Criteria	PLS	QPLS	EB-QPLS	NNPLS	SPL-PLS	MIR-PLS
RMSEC	0.064	0.030	**1.4E-06**	0.050	0.019	0.002
RMSEP	0.297	0.366	0.880	0.566	0.363	**0.263**
*R* ^2^	0.988	0.981	0.892	0.955	0.982	**0.990**
RMSECV	**0.577**	0.678	0.632	0.976	0.674	0.654
MAPECV	**4.113**	4.958	5.131	7.001	4.942	4.634
LVs	4	6	6	7	6	1
Others^a^				15^d^	(2, 5)^b^	0^c^

The bold value means the best performance among different models.

^a^ Other parameters in the model.

^b^ (L, J) used in SPL-PLS, L denotes the degree of polynomial, and J represents the number of knots.

^c^ Number of knots used in MIR-PLS.

^d^ The number of hidden units used in NNPLS.

**FIGURE 7:**
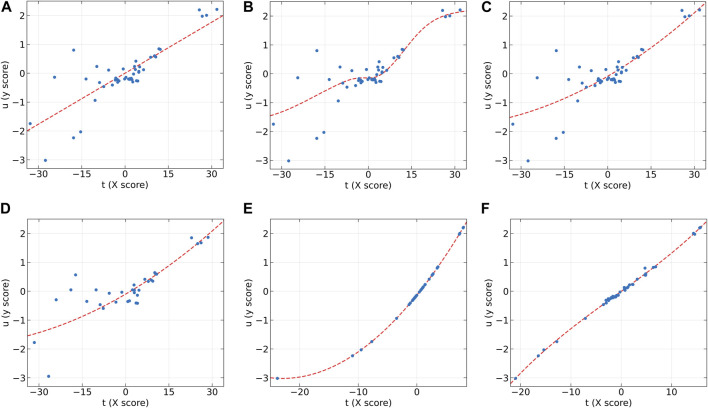
The first LV of the six algorithms on the Beer dataset. **(A)** PLS, **(B)** NNPLS, **(C)** SPL-PLS, **(D)** QPLS, **(E)** EB-QPLS, and **(F)** MIR-PLS.

**FIGURE 8:**
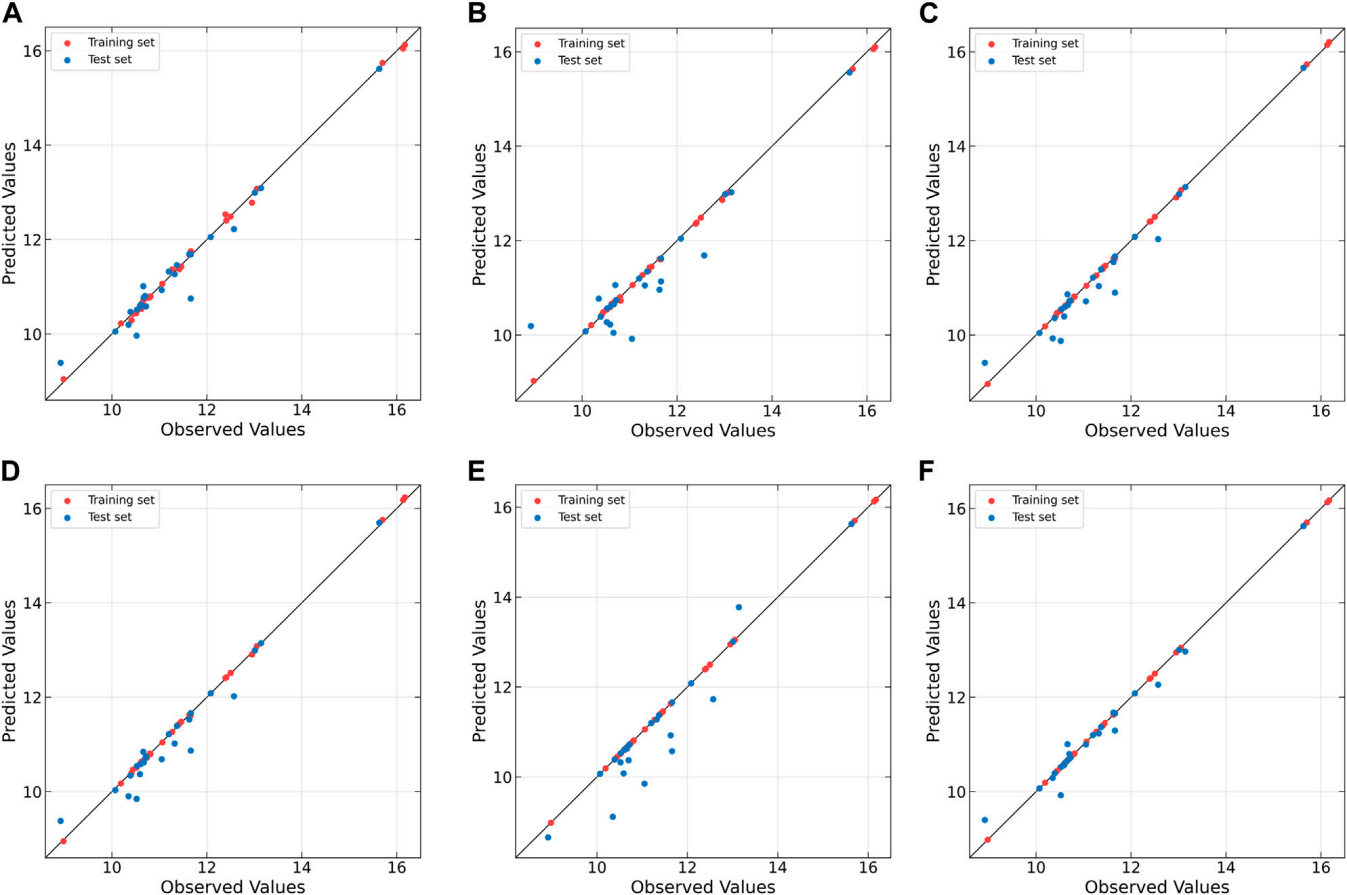
Observed vs. predicted values of original extract concentration for Beer dataset as determined by **(A)** PLS, **(B)** NNPLS, **(C)** SPL-PLS, **(D)** QPLS, **(E)** EB-QPLS and **(F)** MIR-PLS.

EB-QPLS yields the best calibration performance on both Wheat Kernel and Beer datasets. However, it exhibits the poorest RMSEP and *R* ^2^ values on the Beer dataset, indicating that its capacity to generalize is inferior compared to the other models. Figure 7E illustrates that EB-QPLS perfectly fits the inner relation, which is further supported by its observed/predicted plot (Figure 8E) for calibration and test sets. Nevertheless, a closer examination of the test set reveals significant deviations in data points, indicating clear signs of over-fitting. As mentioned earlier, EB-QPLS updates the weights w based on MLR, which explains why it yields very poor validation results, as reported in the literature (Lavoie et al., 2019).

From Table 7, it is evident that NNPLS outperforms PLS in terms of calibration performance. Figure 7B illustrates that a neural network with a single hidden layer can better model inner relations. Surprisingly, however, the generalization performance of NNPLS does not surpass PLS. On the contrary, NNPLS exhibits even poorer, as shown in Figure 8B. Poor performances of NNPLS could be ascribed to the small size of the Beer dataset, comprising only 53 samples compared with the Wheat Kernel dataset (415 calibration samples). This limited sample size may have hindered the construction of a more robust calibration model, as ANNs typically manifest their advantages with a sufficient number of samples.

SPL-PLS demonstrates optimal performances when employing quadratic polynomial function to construct the inner model. Interestingly, the QPLS exhibits performances comparable to SPL-PLS, as can be seen in Figures 7, 8C. These two approaches rank second only to PLS and MIR-PLS in terms of overall performance. However, it is worth highlighting that SPL-PLS and QPLS require 6 LVs to achieve good calibration performance. In contrast, our proposed MIR-PLS approach achieves similar or superior performance using only 1 LV.

The different performances between PLS and our proposed MIR-PLS can be largely explained by the presence or absence of non-linear relationships between **X** and **y**. On the Beer dataset, the calculated non-linear relationship is weak (Figure 7), which explains why the prediction performance of PLS with 4 LVs is close to that of MIR-PLS. However, since linear PLS solely relies on **X-y** relationships, it requires more LVs to approximate MIR-PLS in terms of predictive performance. Figures 7, 8F also demonstrate that MIR-PLS enhances predictive performances for both calibration and test sets, indicating that MIR-PLS has better inner relation fitting capability and calibration performance than the other five models. Compared with EB-QPLS, MIR-PLS is less prone to over-fitting. It yields desirable predictive performance while maintaining excellent calibration performance. However, the Wilcoxon signed rank test (Table 6) shows that the differences between MIR-PLS and other models are not as statistically significant as in the Wheat Kernel dataset.

## 6 Conclusion

This paper aims to propose a new predictive model to deal with complex dose-effect relationship data of CM. Although standard PLS has certain advantages in solving problems such as limited observations and correlated inputs in CM data, it may exhibit poor performances when modeling non-linear relationships. Based on existing methodologies, we proposed a novel NL-PLS based on monotonic inner relations, namely, MIR-PLS. This algorithm employs a piecewise non-linear mapping function to establish non-linear relationships. Moreover, a new WUS is designed to improve predictive performance further. The performance of MIR-PLS was evaluated with three different types of datasets and compared with five well-known PLS variants. On the CM dose-effect relationship datasets, MIR-PLS exhibited outstanding performances in handling non-linear relationships and also performed well with linear relationships. On the NIR datasets, MIR-PLS did not yield the best calibration performances. Nevertheless, in terms of predictive performances, our MIR-PLS algorithm outperformed the others. Even in the presence of multicollinear variables in **X**, this method effectively reduced the risk of over-fitting, demonstrating its reliability and robustness.

## Data Availability

The data analyzed in this study is subject to the following licenses/restrictions: The dose-effect relationship data of Chinese Medicine in this manuscript cannot be shared at this time because it is part of an ongoing study. The Wheat Kernel dataset is available at https://ucphchemometrics.com/datasets/, and the Beer dataset is publicly available at https://www.kaggle.com/datasets/robertoschimmenti/beer-nir/. Requests to access these datasets should be directed to BN, ncunb@163.com.
